# Case 2/2017 - 56-Year-Old Male with Refractory Heart Failure, Systemic Arterial Hypertension and Aortic Valve Stenosis That Led to Heart Transplantation

**DOI:** 10.5935/abc.20170058

**Published:** 2017-05

**Authors:** Desidério Favarato, Paulo Sampaio Gutierrez

**Affiliations:** Instituto do Coração (InCor) HC-FMUSP, São Paulo, SP - Brazil

**Keywords:** Heart Failure, Hypertension, Aortic Valve Stenosis, Heart Transplantation

The patient is a 56-year-old male, born and living in São Paulo, hospitalized due to heart failure decompensation and submitted to heart transplantation.

His symptoms started at the age of 51 years, with dyspnea on moderate exertion, which evolved in 3 months to dyspnea on minimum exertion and orthopnea, and wheeze. The patient required hospitalization for clinical compensation. After discharge, he was referred for treatment at InCor. His discharge prescription included daily furosemide 40 mg, captopril 100 mg, spironolactone 25 mg, and aminophylline 200 mg.

The patient smoked (40 pack-years) and had systemic arterial hypertension. His parents had died due to stroke.

On his first medical visit, one month after that hospitalization, his complaints remained similar to those at the time of hospitalization.

His physical examination (Jul 23, 2008) revealed: weight, 73.6 kg; height, 1.58 m; body mass index, 29.5 kg/m^2^; pulse rate, 76 bpm; right upper limb and right lower limb blood pressures, 148/96 mm Hg and 150/100 mmHg, respectively. His lung auscultation showed no crepitant rales, and his heart auscultation revealed low cardiac sounds with no murmur. His abdominal exam was normal. There was neither lower limb edema, nor signs of increased jugular venous pressure, and his pulses were palpable and symmetrical.

The X ray revealed marked cardiomegaly.

The ECG (Jul 18, 2008) showed: sinus rhythm; heart rate, 67 bpm; PR, 163 ms; QRS duration, 96 ms; QTc, 455 ms; overload of left chambers and secondary changes of ventricular repolarization (Figure1).

**Figure 1:**
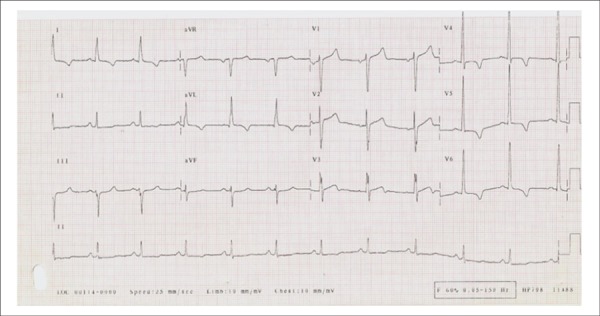
ECG: Sinus rhythm, left atrial and left ventricular overload with strain.

His laboratory tests were as follows: hemoglobin, 14.4 g/dL; hematocrit, 44%; red blood cells, 5,000,000/mm^3^; leukocytes, 11,400/m^3^; uric acid, 9 mg/dL; glucose, 105 mg/dL; creatinine, 1.05 mg/dL; total cholesterol, 266 mg/dL; HDL-C, 35 mg/dL; LDL-C, 153 mg/dL; triglycerides, 412 mg/dL; potassium, 4.6 mEq/L; sodium, 139 mEq/L; and normal urinalysis.

The following diagnoses were established: hypertensive heart disease, obesity, glucose intolerance, hypertriglyceridemia and hyperuricemia.

The echocardiogram (Dec 2, 2008) revealed the following diameters: aorta, 37 mm; left atrium, 44 mm; and left ventricle (diastole/systole), 68/57 mm. The ejection fraction (Teicholz) was 33%, and there was marked diffuse hypokinesia. The septal and posterior wall thickness was 10 mm. The aortic valve showed mild fibrocalcification of its leaflets, maximum and mean transvalvular gradients of 27 mmHg and 17 mmHg, respectively, with estimated valvular area of 1.4 cm^2^, mild stenosis being then considered.

Myocardial MIBI 99mTc scintigraphy with dobutamine (Jan 2009) revealed mild fixed low uptake in the inferior wall, and dilatation and diffuse hypokinesia of the left ventricle with ejection fraction of 27% (Figure 2).

**Figure 2:**
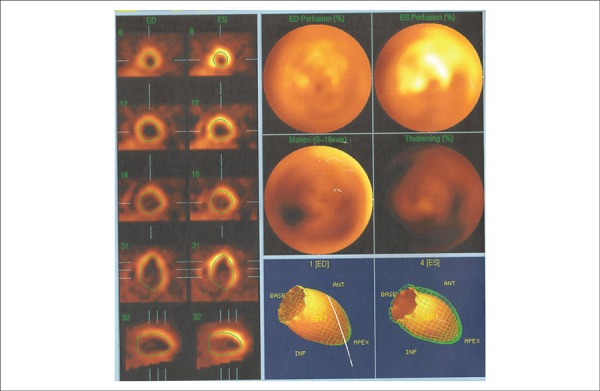
Nuclear ventriculography (gated SPECT scan): Diffuse left ventricular hypokinesia.

The ECG at peak administration of dobutamine, with heart rate of 166 bpm, revealed ST-segment depression, attributed to previous repolarization changes, consequent to left ventricular hypertrophy (Figures 3 and 4).

**Figure 3:**
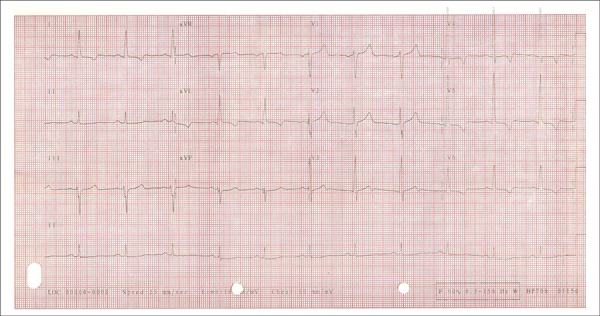
Resting ECG: Ventricular repolarization changes (left leads: inverted T waves).

**Figure 4:**
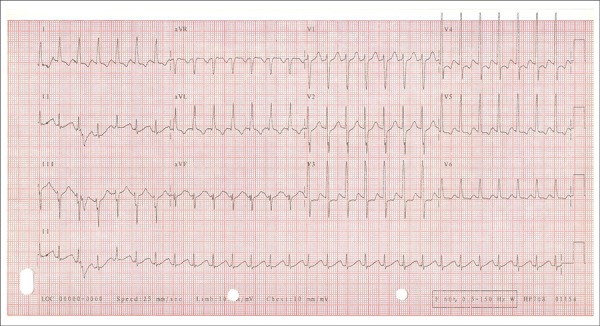
ECG at peak exertion: Heart rate of 167 bpm, ST-segment depression of 1 mm.

Diuretic dynamic renal scintigraphy with DTPA 99mTc showed no change in renal perfusion, clearance or size.

Spirometry revealed mild obstructive disorder, which improved with bronchodilator use.

The patient progressed with dyspnea on moderate exertion and an episode of syncope preceded by chest pain. Coronary cineangiography showed no obstructive lesion. The left circumflex artery was small and the right coronary artery was dominant (Dec 15, 2009). The following drugs were prescribed: daily enalapril, 40 mg; carvedilol, 12.5 mg; furosemide, 40 mg; spironolactone, 25 mg; propatylnitrate, 30 mg; simvastatin, 40 mg; acetylsalicylic acid, 100 mg; and salbutamol, 6 mg.

The patient sought the emergency unit because of dyspnea worsening for 15 days, being then on minimum exertion and orthopnea, with lower limb edema and episodes of chest pain, some of which lasted longer in the last 3 days. He related that worsening to interruption of the medication.

The physical examination (Jan 21, 2014) revealed: pulse rate, 84 bpm; blood pressure, 100/70 mm Hg; normal lung and heart auscultations; normal abdominal exam; and lower limb edema, +++/4+.

The chest X ray (Jan 21, 2014) showed increased pulmonary vascular bed and global cardiomegaly (Figure 5).

**Figure 5:**
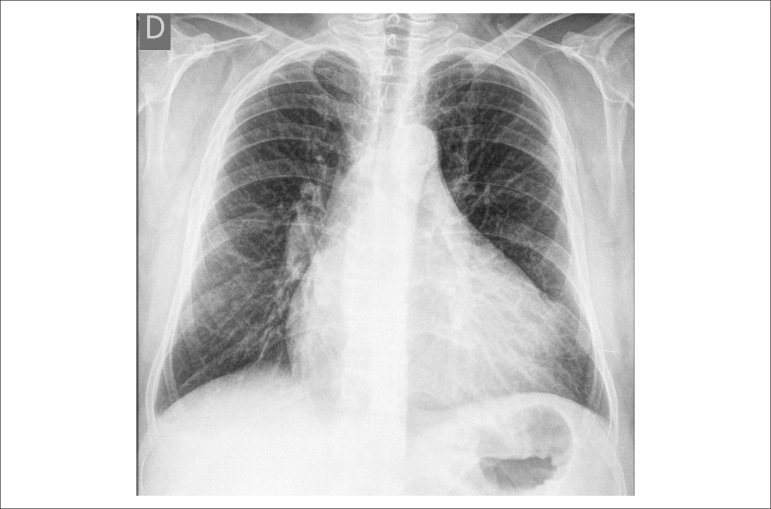
Chest X ray (PA). Pulmonary fields with signs of congestion: vascular bed inversion and apparent fissure; marked global cardiomegaly.

The laboratory tests revealed: increased levels of myocardial lesion markers (CK MB, 12.43 ng/mL; troponin I, 0.38 ng/mL), which decreased in the following measurements; red blood cells, 5,300,000/mm^3^; hemoglobin, 15 g/dL; hematocrit, 49%; leukocytes, 7,540/mm^3^ (61% neutrophils, 1% eosinophils, 1% basophils, 32% lymphocytes, and 5% monocytes); platelets, 183,000/mm^3^; urea, 108 mg/dL; creatinine, 1.73 mg mg/dL (glomerular filtration 44 mL/min/1.73 m^2^); TSH, 7 *µ*UI/mL; sodium, 134 mEq/L; potassium, 4.0 mEq/L; PT (INR), 1.5; and APTT (rel), 1.07.

The new echocardiographic assessment (Jan 27, 2014) showed the following diameters: aorta, 35 mm; left atrium, 55 mm; right ventricle, 34 mm; left ventricle (diastole/systole), 76/72 mm. Ejection fraction was 20%, and septal and posterior wall thickness was 9 mm. The left ventricle showed marked diffuse hypokinesia, and the right ventricle, moderate hypokinesia. The aortic valve was moderately fibrocalcific, with reduced mobility of its leaflets, and maximum and mean transvalvular gradients of 28 mm Hg and 18 mm Hg, respectively. The estimated systolic pulmonary pressure was 50 mm Hg.

One week after admission, the laboratory reassessment revealed worse kidney function with creatinine of 2.24 mg/dL (glomerular filtration of 32 mL/min/1.73m^2^) and urea of 119 mg/dL.

The patient had pneumonia, arterial hypotension and low cardiac output, the last two persisting even after pneumonia treatment. The patient received vasoactive amines and intra-aortic balloon for circulatory support. Heart transplantation was indicated.

His new laboratory assessment 1.5 month after admission revealed: hemoglobin, 11.6 g/dL; hematocrit, 36%; leukocytes, 9,280/m^3^; platelets, 90,000/mm^3^; urea, 77 mg/dL; creatinine, 1.44 mg/dL (glomerular filtration 54 mL/min/1.73 m^2^); AST, 39 U/L; ALT, 26 U/L; alkaline fosfatase, 142 U/L; gamma GT, 332 U/L.

The patient underwent orthotopic heart transplantation (Mar 18, 2014).

## Clinical aspects

The 56-year-old male patient, who smoked and had arterial hypertension and hypertriglyceridemia, developed heart failure with dilatation of heart chambers and severe systolic dysfunction. His echocardiography showed low-gradient aortic stenosis.

According to the Brazilian Society of Cardiology III Guideline on Heart Failure, a patient with signs and symptoms of heart failure should undergo some tests to characterize the heart failure, such as resting electrocardiography, chest X ray, echocardiography and BNP measurement. Then, laboratory and invasive tests, such as coronary cineangiography and cardiac biopsy, should be performed for etiological diagnosis.^1^

In the case here reported, there were neither epidemiological data suggestive of Chagas disease, nor conduction disorders usually found in that disease, such as right bundle-branch block and left anterior block, whose prevalence is three times greater than that in the general population.^2,3^

Another etiology to remember is rheumatic heart disease, because mild aortic stenosis was detected on echocardiography. In rheumatic disease, the mitral valve is most commonly affected, followed by double mitral-aortic impairment and isolated aortic valve impairment. Although there was no report of an acute rheumatic fever episode during childhood, that often passes unnoticed by the patients with that heart valve disease. Against that diagnosis is the age of clinical manifestation, usually around 30 years, although the age may range from 20 years to 50 years.^4,5^

Persistent rheumatic carditis can be the cause of heart failure with ventricular dilatation. However, the age group is younger, from 5 years to 20 years, being a cause of diagnostic confusion with infective endocarditis and not with heart failure etiology.^6^

There were risk factors for coronary heart disease, such as arterial hypertension, low HDL-cholesterol levels, hypertriglyceridemia and smoking, predictors of acute ischemic coronary events at a younger age.^7,8^

The present patient had a history of neither acute myocardial infarction nor typical angina. The echocardiogram revealed no segment deficiency of contractility, but identified diffuse hypokinesia. Scintigraphy evidenced a mild fixed uptake reduction in the inferior wall, which is not rare in patients with dilated cardiomyopathy.^9^

Even adding the changes in scintigraphy to the electrocardiographic ones, the later attributable to left ventricular overload signs, the chances of ischemic cardiomyopathy would be low. However, one should rule out the diagnosis of any treatable cause of heart failure, such as coronary artery disease. Considering our patient's clinical findings, coronary cineangiography was properly indicated, although it resulted normal.

In addition, non-rheumatic aortic valve stenosis could explain all the patient's clinical findings. Although the aortic valve stenosis is considered to have hemodynamic repercussion if the valvular area is equal to or smaller than 1 cm^2^ and the mean gradient is greater than 40 mm Hg, the clinical entity called low-flow, low-gradient aortic stenosis has been recently increasingly studied. Its most common presentation is marked left ventricular dilatation and very low ejection fraction, and some authors have reported its prevalence ranging from 5% to 10% of patients with marked aortic valve stenosis. The prognosis is very poor, with 3-year mortality of 50% for patients on drug treatment, and of 6% to 30% for patients undergoing surgery. Thus, the precise assessment of the grade of stenosis and of myocardial dysfunction is essential to determine those patients' treatment. When the diagnosis is uncertain, some diagnostic methods can be used, such as stress Doppler echocardiography with dobutamine to assess low flow reserve (lack of minimal 20% increase in left ventricular systolic volume). Another diagnostic method is to measure the calcium score of the heart valve on cardiac tomography, usually greater than 1650 Agastston.^10^

Against that diagnosis is the patient's heart valvular area greater than 1.2 cm^2^ and his age, because, on average, individuals with that type of stenosis are older than 70 years.^11^

Because the patient has arterial hypertension, hypertensive heart disease cannot be ruled out as responsible for the patient's clinical findings.

In the Framingham study, in 91% of the cases with heart failure, arterial hypertension preceded that condition. Hypertension doubled the incidence of heart failure in men and tripled it in women. In Brazil, arterial hypertension associated with coronary artery disease is the most frequent cause of heart failure.^12^

The incidence of heart failure is proportional to blood pressure levels, to age and to hypertension duration. Blood pressure control can decrease the incidence of heart failure by 50%.^13,14^

Untreated arterial hypertension causes changes in the sympathetic and renin-angiotensin-aldosterone systems, which lead to hypertrophy, followed by myocyte apoptosis and autophagy, proliferation of fibroblasts, interstitial collagen accumulation, and, eventually, adverse remodeling and pump failure.^15-18^

The diagnosis of dilated cardiomyopathy is limited in the present case, because of the presence of known causes of heart failure, such as arterial hypertension and aortic valve disease.

Regarding the final cardiac decompensation, it is worth noting its major causes: drug non-adherence, cardiac arrhythmias, and disease progression and complications, such as pulmonary infection or thromboembolism. In the present case, there was infection, and neither thromboembolism nor disease progression can be ruled out. (Desidério Favarato, MD)

Diagnostic hypothesis: heart failure due to cardiopathy with ventricular dilatation. Etiology: hypertensive heart disease or aortic valve disease, non-rheumatic aortic stenosis. (Desidério Favarato, MD)

## Anatomopathological examination

**Figure 6:**
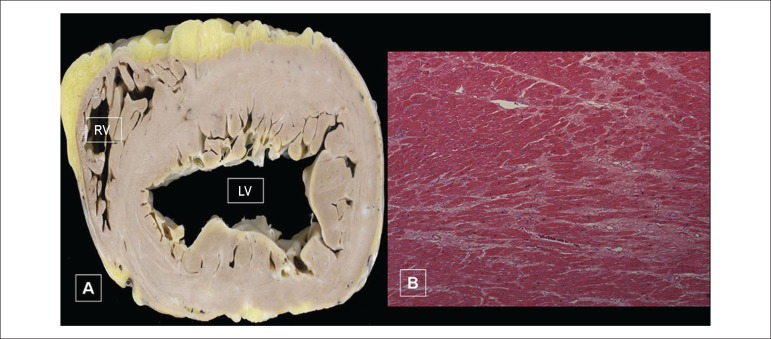
On gross examination, cross-section of the heart (distorted by fixation) (A) at ventricular level, showing dilatation of the cavities, with normal to mildly increased wall thickness. Note the presence of small white areas in the left ventricular wall, corresponding to fibrosis, also identified on the microscopic exam (B) as lighter areas amidst the darkly stained myocardium. (Hematoxylin-Eosin; x5). RV: right ventricle; LV: left ventricle.

**Figure 7:**
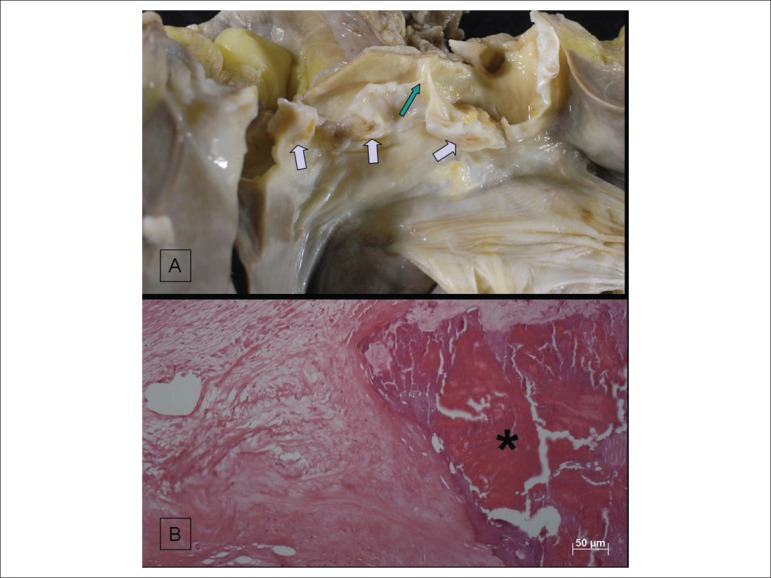
A) Opened aortic valve showing calcification nodules in its three leaflets (purple arrows). There is no commissural fusion (green arrow). B) Microscopic section of the aortic valve with areas of dense calcification (asterisk). Note the absence of inflammatory cells. (Hematoxylin-Eosin; x5).

The formalin-fixed explanted heart missed part of the left atrium and weighed 580 g. It showed dilatation of the ventricular cavities, and focal areas of fibrosis (Figure 6A). The microscopic study evidenced hypertrophy of myocardial fibers and interstitial fibrosis (Figure 6B). On gross examination, the aortic valve was thickened and calcified, without commissural fusion (Figure 7A). The other valves, pericardium and coronary arteries showed no significant changes. After decalcification, the microscopic study of the aortic valve evidenced fibrous thickening, dense calcification and no inflammatory signs (Figure 7B). **(Paulo Sampaio Gutierrez, MD)**

**Anatomopathological diagnoses:**
- Calcified aortic valve disease
- Hypertensive cardiomyopathy
- Dilatation of the cardiac chambers with hypertrophy of myocardial fibers and areas of interstitial fibrosis **(Paulo Sampaio Gutierrez, MD)**

## Comment

The major question in this case is the cause of heart failure, in particular the causative role aortic valve disease might have played.

The anatomopathological exam is not ideal to assess heart valve dysfunction, because the heart is analyzed without movement and stiffened by fixation. However, the significant calcification suggests that heart valve disease might have contributed significantly to heart failure. On the other hand, the patient is hypertensive, and, thus, hypertensive cardiomyopathy should not be ruled out.

Thus, both processes - systemic arterial hypertension and calcified aortic valve disease - should be considered to play a role in the installation of cardiac dysfunction.

Regarding the cause of valve heart disease, the lack of a clinical history, of mitral valve impairment, of aortic valve commissural fusion and of inflammatory cells on the microscopic study indicates this is not consequent to rheumatic disease. The diagnosis to be considered is "degenerative" valve disease, and it is worth noting, however, that the patient's age is under the age group in which that lesion usually causes symptoms sufficiently severe to require surgery: in a series,^19^ less than 6% of the men with tricuspid aortic valve (as our patient) underwent surgery before the age of 60 years, and none before the age of 50, when our patient already had, on echocardiography, moderate stenosis. A European multicenter study has reported a mean age of 69 years for patients with aortic stenosis.^20^ **(Paulo Sampaio Gutierrez, MD)**

**Section editor:** Alfredo José Mansur (ajmansur@incor.usp.br)

**Associated editors:** Desidério Favarato (dclfavarato@incor.usp.br)

Vera Demarchi Aiello (vera.aiello@incor.usp.br)
